# Graded isavuconazonium sulfate challenge in a patient with hypersensitivity to fluconazole

**DOI:** 10.1016/j.idcr.2025.e02355

**Published:** 2025-09-08

**Authors:** Mark Biagi, Michael Pierce, Wendy Harney, Otavio Pereira-Rodrigues, Geoffrey Tsaras

**Affiliations:** a Department of Pharmacy, UW Health Swedish American Hospital, Rockford, IL, USA; b College of Pharmacy, University of Illinois at Chicago, Rockford, IL, USA; c Department of Pharmacy, UW Health University Hospital, Madison, WI, USA; d Department of Internal Medicine, Section of Infectious Diseases, UW Health Swedish American Hospital, Rockford, IL, USA; e College of Medicine, Department of Infectious Diseases, University of Illinois at Chicago, Rockford, IL, USA

**Keywords:** Candidiasis, Fluconazole, Hypersensitivity, Isavuconazonium sulfate, Isavuconazole, Rechallenge

## Abstract

Hypersensitivity reactions to triazole antifungals are uncommonly reported and cross-reactivity between these agents is poorly understood. We describe our experiences in a patient who previously developed a full body rash after receiving fluconazole who was initially successfully challenged and treated with isavuconazole without experiencing any adverse effects and was later separately re-challenged and treated successfully with a full-course of isavuconazole 8 months later. She received a third course of isavuconazole approximately 10 months after her second course, and developed rash, chest tightness, dyspnea, and facial flushing.

## Introduction

Hypersensitivity reactions to triazole antifungals are infrequently encountered, and cross-sensitivity between these agents is poorly understood. Available literature on the subject is primarily limited to case reports with often conflicting results [1], [2], [3], [4], [5], [6], [7]. We report our experience in a patient with a history of diffuse full-body rash following exposure to fluconazole who was initially successfully challenged and treated with isavuconazole but later developed signs and symptoms of a hypersensitivity reaction during a later course of therapy.

## Case presentation

A 55-year-old female was seen by her otolaryngologist for recurrent oral, esophageal, and laryngeal candidiasis causing dysphagia and dysphonia for the past two years. The underlying cause of her recurrent infections was multifactorial and believed to be a consequence of excessive use of antibiotics for respiratory infections, use of inhaled corticosteroid therapy for maintenance therapy of asthma, and intermittent use of oral corticosteroids for acute asthma exacerbations. She previously received fluconazole on multiple occasions without incident but developed a blotchy full body and facial rash associated with severe itching that started the morning after the first dose of her last course approximately three months earlier. She was advised over the telephone to take an oral antihistamine by her primary care provider but was not seen in-person for medical treatment. Following this, she was subsequently prescribed oral nystatin but continued to have persistent symptoms and was referred to our infectious diseases clinic. She had a positive fungal culture for *Candida albicans* from an oral cavity sample approximately one month prior to her visit. It was decided to pursue desensitization to a triazole to avoid placing a central line. Since the patient was taking rimegepant for migraines, strong CYP3A4 inhibitors (itraconazole, posaconazole, and voriconazole) were avoided and isavuconazole, a moderate CYP3A4 inhibitor, was selected.

The patient was eventually admitted to the intensive care unit to undergo a graded challenge to intravenous isavuconazonium sulfate, as summarized in Table 1. Intensive care admission is routinely performed for all patients undergoing desensitization or graded challenges at our institution to ensure careful monitoring for signs or symptoms of a hypersensitivity reaction and to ensure titrations are performed in a timely manner. No pre-medications were administered to the patient prior to the start of the graded challenge. A single 250 mL infusion bag containing isavuconazonium sulfate 372 (equivalent to isavuconazole 200 mg) diluted in 5 % dextrose was prepared. The infusion rate started at 25 mL/hr and subsequently increased by 25 mL/hr every 15 min until it reached a final infusion rate of 225 mL/hr. This graded challenge protocol was independently created due to the lack of a standardized protocol in the literature and was intentionally developed to have a consistent titration of the infusion rate (25 mL/hr) after each step to decrease the risk of ordering or administration errors. The process was completed successfully with no signs or symptoms of a hypersensitivity reaction, and the patient was discharged home the following day. Five days post-discharge, the patient was seen in the clinic for routine follow-up where she reported no symptoms of candidiasis or side effects.

**Table 1:** Isavuconazole graded challenge protocol^A^.

**Step**	**Infusion rate (mL/hour)**^B^	**Isavuconazole dose** **(mg)**	**Cumulative isavuconazole dose** **(mg)**
1	25	5	5
2	50	10	15
3	75	15	30
4	100	20	50
5	125	25	75
6	150	30	105
7	175	35	140
8	200	40	180
9	225	20	200

A Preparation was performed by reconstituting a single vial of isavuconazonium sulfate 372 mg in 5 mL of sterile water. The reconstituted solution was subsequently diluted in 250 mL of 5 % dextrose.

B For steps 1–8, the infusion rate was titrated every 15 min.

Approximately eight months later, the patient experienced an asthma exacerbation and was prescribed inhaled fluticasone propionate. Despite aggressive oral rinsing, she developed dysphagia and dysphonia associated with thrush. The patient was instructed she may need to be readmitted for a repeat graded challenge to isavuconazole but strongly preferred to trial the drug in the outpatient setting without graded challenge. After discussion and careful consideration, she was prescribed oral isavuconazonium sulfate in the outpatient setting without graded challenge. At her follow-up appointment three weeks later, she reported no side effects and complete resolution of her dysphagia and dysphonia.

Ten months after this episode, the patient developed another recurrent episode of dysphagia and dysphonia associated with thrush. She was again prescribed oral isavuconazonium sulfate in the outpatient setting without graded challenge. Five days into her treatment course, she contacted the clinic stating that she had developed a new rash on day 2 of therapy that did not resolve with diphenhydramine, followed by development of chest tightness and mild dyspnea over the next few days that necessitated the use of her albuterol inhaler. She denied hives and facial/oral swelling. It was unclear whether the patient’s pulmonary symptoms were related to a hypersensitivity reaction or her underlying asthma. Out of an abundance of caution, the patient was instructed to discontinue isavuconazole immediately and follow-up with her dermatologist. A peripherally inserted central catheter was eventually placed and the patient was treated with micafungin.

## Discussion

Hypersensitivity reactions to triazoles are uncommon and cross-reactivity between these agents is poorly understood. For example, among seven published cases evaluating potential cross-reactivity between fluconazole and itraconazole, three cases concluded cross-reactivity between these agents [1], [3], [7], whereas the remaining four cases reported a lack of cross-reactivity [2], [4], [5], [6].

Data on the cross-reactive potential of isavuconazole with other triazoles is even more scarce. Morales and colleagues previously reported the successful use of a graded isavuconazole introduction in a hepatic transplant patient with a history of voriconazole-associated angioedema [8]. In a separate report, a patient who had previously developed a full-body pruritic, maculopapular rash attributed to itraconazole tolerated isavuconazole using the protocol developed by Morales et al. and premedication with cetirizine [9]. In contrast, a 51-year-old male with pulmonary cryptococcosis, who developed bilateral pruritic and erythematous lesions following two doses of fluconazole, experienced rash recurrence following an oral graded isavuconazole challenge [10]. Our patient tolerated isavuconazole without graded challenge during her second course of therapy but later developed rash, dyspnea, and chest tightness when rechallenged to a third course of isavuconazole therapy. However, we acknowledge that the delayed development of dyspnea and chest tightness may be attributed to her underlying asthma and may not represent an IgE-mediated reaction to isavuconazole. Our experiences reported here highlight the current poor understanding of triazole hypersensitivity reactions and cross-reactivity.

In contrast to the protocol used by Morales et al., advantages of our graded challenge protocol are a shorter time to completion (1 day versus 5 days), potentially leading to a shorter length of stay, requires availability of only the intravenous formulation, uses only one intravenous vial, and involves the preparation of only one infusion bag instead of three [8]. However, it should be noted that the reaction our patient experienced with fluconazole was non-life threatening unlike the reaction to voriconazole experienced by the patient reported by Morales et al. Additionally, considering our patient had previously tolerated fluconazole, we cannot rule out that our patient’s reaction to fluconazole was triggered by an excipient since dispensing records were not available to confirm whether she received the same fluconazole formulation produced by the same manufacturer each time.

Although isavuconazole was initially prescribed over other triazoles due a lower risk of drug interactions, our experiences suggest that isavuconazole may be a useful agent in the setting of patients who develop hypersensitivity reactions to other triazoles and provide clinicians with a graded challenge protocol that can be complete in a timely manner.

## CRediT authorship contribution statement

**Michael Pierce:** Writing – review & editing, Writing – original draft. **Mark Biagi:** Writing – review & editing, Writing – original draft, Conceptualization. **Wendy Harney:** Writing – review & editing, Writing – original draft. **Otavio Pereira-Rodrigues:** Writing – review & editing, Writing – original draft. **Geoffrey Tsaras:** Writing – review & editing, Writing – original draft.

## Declaration of Competing Interest

The authors declare that they have no known competing financial interests or personal relationships that could have appeared to influence the work reported in this paper.
